# Active visual search in naturalistic environments reflects individual differences in classic visual search performance

**DOI:** 10.1038/s41598-023-27896-7

**Published:** 2023-01-12

**Authors:** Thomas L. Botch, Brenda D. Garcia, Yeo Bi Choi, Nicholas Feffer, Caroline E. Robertson

**Affiliations:** 1 Department of Psychological and Brain Sciences, Dartmouth College, Hanover, NH 03755 USA; 2 Department of Computer Science, Dartmouth College, Hanover, NH 03755 USA; 3 Department of Computer Science, Stanford University, Stanford, CA 94305 USA

**Keywords:** Psychology, Human behaviour

## Abstract

Visual search is a ubiquitous activity in real-world environments. Yet, traditionally, visual search is investigated in tightly controlled paradigms, where head-restricted participants locate a minimalistic target in a cluttered array that is presented on a computer screen. Do traditional visual search tasks predict performance in naturalistic settings, where participants actively explore complex, real-world scenes? Here, we leverage advances in virtual reality technology to test the degree to which classic and naturalistic search are limited by a common factor, set size, and the degree to which individual differences in classic search behavior predict naturalistic search behavior in a large sample of individuals (N = 75). In a naturalistic search task, participants looked for an object within their environment via a combination of head-turns and eye-movements using a head-mounted display. Then, in a classic search task, participants searched for a target within a simple array of colored letters using only eye-movements. In each task, we found that participants’ search performance was impacted by increases in set size—the number of items in the visual display. Critically, we observed that participants’ efficiency in classic search tasks—the degree to which set size slowed performance—indeed predicted efficiency in real-world scenes. These results demonstrate that classic, computer-based visual search tasks are excellent models of active, real-world search behavior.

## Introduction

Locating an object in a cluttered environment is a ubiquitous visual behavior. The mechanisms by which humans accomplish visual search have been comprehensively studied in traditional computer-based settings using both artificial arrays^1^ and complex scene images^2^. Yet, little is known about whether the principles of visual search revealed by these studies extend to active, self-directed exploration in real-world environments, and whether individual performance in both traditional and naturalistic contexts is limited by common factors.

Classic, computer-based studies have identified numerous factors that govern visual search performance^3^. For example, search is limited by the similarity between the visual features of a target (e.g., color, shape, size) and the array of distractors in which it is embedded^4^. A key component of these studies is the use of minimalistic, simplified stimulus arrays, which allow experimenters to systematically manipulate one factor of interest (e.g., color), while controlling for others (e.g., shape, size), and to measure the impact of this isolated factor on performance. This approach has provided insights into the mechanisms underlying visual search and inspired multiple formal and conceptual models of the behavior^1,5,6^. Further, these models underpin frameworks for understanding diverse cognitive processes including attention^4,6–9^, reward^10,11^, and decision-making^12^.

However, the computer-based approach contains two key drawbacks that limit generalization to real-world search behavior^13–15^. First, artificial stimuli lack the complex visual statistics and structural cues present within real-world scenes^14^. Recent computer-based studies investigating search in complex scene images demonstrate that the structure of the visual environment supplements attentional guidance beyond basic factors probed in paradigms with minimalistic stimuli^16,17^ by engaging episodic and semantic memory^18,19^ and guiding eye-movements to visual targets^20,21^. Second, computer-based approaches engage working memory differently from active, immersive contexts^22^. During active exploration, working memory operates across multiple spatial reference frames to guide attention^23^. Thus, naturalistic paradigms present a valuable opportunity to validate models of human behavior derived in traditional laboratory settings and extend these models to the conditions and demands of everyday life^24,25^.

Indeed, decades of research have established many connections between visual search in laboratory settings and in real-world environments^26–31^. In particular, researchers have characterized visual search performance in multiple professional contexts including radiology^32–34^, airport security^35,36^, and driving^30^. These studies have revealed numerous features of computer-based visual search that translate to everyday settings. For example, these studies have shown that experienced radiologists are both faster and more accurate at detecting abnormalities in medical images than naïve observers^37–39^. In the context of airport security, individual differences in search speed and accuracy measured on a computer-based app have been shown to predict target detection at TSA checkpoints^40^. Interestingly, not all aspects of the laboratory are paralleled in real-world environments. For example, because radiologists and airport security officers encounter targets at lower rates in occupational settings, as compared with laboratory paradigms, error rates (misses) are relatively higher and false alarm rates lower in these occupational settings, regardless of expertise^28,38^. Together, these studies show important parallels of visual search performance across computer-based and real-world contexts.

Virtual reality (VR) offers complementary opportunities to investigate visual behavior in naturalistic contexts. Similar to real-world settings, head-mounted VR displays allow researchers to study search in active conditions, where working memory can guide search across spatiotopic reference frames^23,41^. However, in contrast to real-world settings, VR enables researchers to present diverse sets of stimuli with ease, manipulate specific environmental features of these stimuli, and explore the contributions of these factors to visual search performance. Recent studies have investigated active visual search behavior using head-mounted VR^42–44^. These studies again highlight the importance of environmental structure (e.g., scene layout, semantics) in shaping active visual search strategies^45–48^. However, these studies have largely employed minimalistic, computer-rendered virtual environments as stimuli, where the experimenter can manipulate scene content and structure to identify regularities that facilitate attentional guidance in active settings. Because such rendered stimuli do not contain real-world visual content, these studies are subject to the first limitation of the classic paradigms described above: they cannot address the degree to which the statistical regularities of real-world scenes impact search in active, naturalistic settings.

Here, we leverage advances in VR technology to study the common factors limiting visual search in classic, computer-based paradigms and immersive scenes with real-world visual content. We specifically focused on one key factor that limits search performance in classic studies, set size: the number of items within a visual array. Increasing set size impairs search performance in both artificial arrays^49,50^ and images of complex scenes^21,51,52^. However, it remains unclear whether set size effects analogously limit behavioral performance during active exploration of real-world environments, where environmental structure and memory are available to aid attentional guidance^53,54^. Further, to our knowledge, whether individual differences in search efficiency in artificial displays predict naturalistic search performance in real-world environments has never been explored.

Thus, our study aimed to answer two questions: (1) does set size limit both classic and naturalistic search, and (2) is search efficiency on classic, computer-based search tasks predictive of active search performance in real-world scenes? Participants (N = 75) completed two tasks: (1) a classic, computer-based conjunctive search paradigm with arrays varying in set size and (2) a naturalistic, VR-based search paradigm with immersive, real-world environments varying in levels of visual clutter^55^. In both tasks, we characterized the impact of set size on visual search performance. We also tested whether participants’ search efficiency was related across the two paradigms (classic and naturalistic).

## Methods

### Participants

75 adults participated in two experiments (N = 49 females; mean age 21.55 + /− 3.31 STD years). Participants were recruited based on (1) having normal or corrected-to-normal vision and no colorblindness, (2) having no neurological or psychiatric conditions, and (3) having no history of epilepsy. We selected our sample size based on comparable studies^46,47^, and no participants were excluded from the analysis. Written consent was obtained in accordance with the Declaration of Helsinki via a protocol approved by the Dartmouth College Ethics Committee for the Protection of Human Subjects (CPHS).

### Remote data collection

Participants received a standalone head-mounted display (Oculus Quest 2, www.oculus.com, single fast-switch LCD, 1832 × 1920px per eye; ~ 90° field of view; 72 Hz refresh rate) preconfigured with the ManageXR (www.managexr.com) device management software. Experiments were built in Unity version 2018.4.12f1 (www.unity.com) with custom scripts written in C#. Experimental data was collected through a custom data transfer pipeline written in C# and PHP to transmit data from the HMD to lab servers.

### Experiment 1: Naturalistic visual search

#### Exp. 1—Stimuli and set size manipulation

 In the naturalistic search experiment, stimuli consisted of 360° “photospheres” of real-world scenes, sourced from an online photo sharing website (www.flickr.com). We curated 54 photospheres with four criteria to minimize the complications of defining set size in real scenes^52^. First, we selected photospheres of indoor scenes, as outdoor scenes contain few segmented regions which may not be representative of the true set size. Second, we ensured the photospheres did not contain humans to avoid the possibility that humans are a unique object category. Third, we confirmed that each photosphere contained a “singleton” target object: an object that appeared only once inside a given photosphere. Fourth, given the importance of depth to scene processing in early visual areas on the brain^56^, we ensured that all photospheres had comparable depth. To this end, we estimated the depth of each photosphere using the big-to-small (BTS) algorithm^57^.

**Figure 1:**
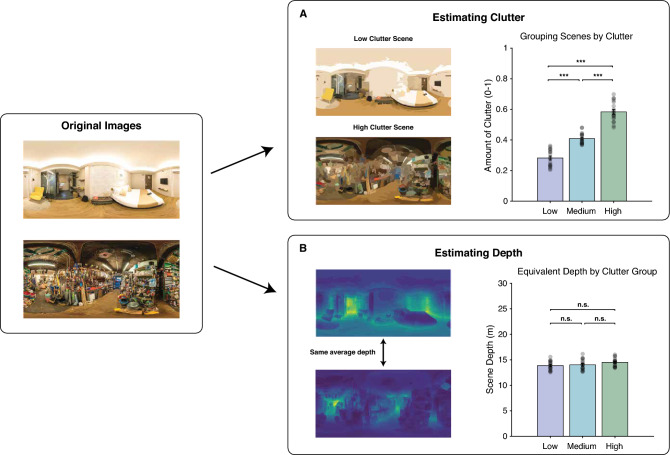
Visual clutter and depth estimation in real-world scene stimuli. (**A**) Example visualizations of visual clutter estimated by the proto-object segmentation algorithm. Photospheres were divided into three bins, and average clutter of each bin significantly differed from the others (*F*_(2,51)_ = 144.7, *p* < 0.001). (**B**) Example visualizations of scene depth estimated by the big-to-small algorithm. The average depth of each clutter bin did not significantly differ (*F*_(2,51)_ = 1.20, *p* = 0.331). In all plots, error bars represent 1 SEM. *p < 0.05, **p < 0.01, ***p < 0.001, n.s. *p* > 0.05.

We adopted the concept of visual clutter as a proxy for set size in real-world scenes^49,55^ and approximated the visual clutter of each photosphere using the proto-object segmentation algorithm^58^. Subsequently, we divided the photospheres into three bins (18 photospheres each) based on the estimated clutter measurements (low, medium, and high clutter) and ensured that the average clutter of each bin significantly differed from the others (Fig. 1A). The average depth of photospheres in each bin did not significantly differ between bins (Fig. 1B).

Target object locations were balanced across photospheres within each clutter bin. For each scene, the yaw of each photosphere was randomly rotated such that the target object was located in one of three quadrants of the immersive environment relative to the participant’s initial facing direction: (1) to the left of the participant, (2) in front of the participant, or (3) to the right of the participant. This resulted in an equal distribution of target object locations relative to the participant across the three possible quadrants (6 photospheres per quadrant), and across the clutter bins (18 photospheres per quadrant).

#### Exp. 1—Paradigm

**Figure 2:**
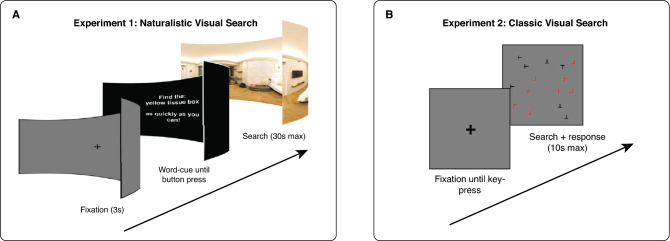
Experimental paradigms. (**A**) Naturalistic visual search paradigm. After a pre-trial fixation, participants were presented with a conjunctive word description of a target object. Subsequently, participants actively searched (e.g., head-turns, saccades, etc.) for the described object inside an immersive photosphere. (**B**) Classic visual search paradigm. After a pre-trial fixation, participants searched for a red T within a cluttered array shown via a head-fixed display.

 On each trial of the naturalistic visual search experiment (54 trials), participants were presented with a photosphere via the head-mounted display (HMD) for a maximum of 30 s, or until the controller trigger was pressed indicating detection of the target object (Fig. 2A; Supplemental Video S1). In all scenes, an occluding wall obstructed the 90° immediately behind the participant such that the 270° in front of the participant was visible. Accordingly, participants were informed that the area behind them would not be visible and instructed to explore the forward, left, and right portions of the photosphere. To mitigate confusion during the real-world visual search task, we informed participants that the target object would always be present inside the virtual environment.

Before each trial, participants were presented with a pre-trial fixation target at screen-center to ensure participants entered each photosphere facing the same direction. Participants were required to align their head-center with the target for 3 s. Subsequently, participants were presented with a conjunctive word cue (e.g., green bottle) describing the target object in the following photosphere. Participants were instructed to “find the target as quickly as possible”. To report the target, participants centered their head on the target (specifically, they centered a light gray circle, which was locked to screen-center, on the target) and pressed the controller trigger. A response was considered correct if the participant’s head coordinate was within a 7.5° visual angle radius from target center when the trigger was pressed, and reaction time was calculated as the time of the trigger press relative to trial start. After pressing the trigger, participants were given feedback on the accuracy of their response. The gray, head-locked circle would turn green if the participant selected the correct object and would turn red if the participant selected an incorrect object. After each trial, participants were returned to a virtual home environment where they were informed of their reaction time and instructed to take a break. A mandatory break occurred after each quarter of the experiment (14 trials) to allow participants to rest their eyes.

At the start of the study, participants were shown a set of instructions orienting them to the task. Following the instructions, participants completed two practice trials to ensure familiarity with the task. Participants were highly accurate during practice trials (mean accuracy: 84%), indicating comprehension of the task.

### Experiment 2: Classic visual search

#### Exp 2—Stimuli and set size manipulation

 In the classic visual search experiment, stimuli consisted of letter arrays, which were presented on a gray background around a central fixation point (Fig. 2B). The letters in the array had two feature dimensions: form (Ts and Ls) and color (red and black). Arrays spanned 25° × 25° visual angle, and letters within the array were randomly distributed around a central fixation point and spaced from others by 2° visual angle. Displays had three potential set size conditions: 5, 15, or 25 letters.

#### Exp 2—Paradigm

 On each trial of the classic conjunctive search task (180 trials), participants were instructed to report the presence/absence of a target letter (a red T) using a keypad. Note, the target letter shared a feature dimension with each type of distractor (black Ts and red Ls). There were two trial types, target present or target absent, which each occurred 50% of the time. On trials without a conjunction target, an additional distractor was added at random.

Each trial lasted for a maximum of 10 s or until a keypress. Before each trial, participants were shown a black fixation cross and required to press a button to start the trial. Participants were instructed to fixate on the cross until trial start, after which point they were free to move their eyes. Participants were instructed to “find the target as quickly as possible” and to “press 4 if the target is present or 6 if the target is absent”. Participant reaction time was calculated as the time of the button press relative to trial start. Following each trial, participants were given feedback on the accuracy of their response (a green check for correct responses and a red X for incorrect responses). A mandatory break occurred every 45 trials to allow participants to rest their eyes.

At the start of the study, participants were shown a set of instructions orienting them to the task. Following the instructions, participants completed a set of practice trials (12 trials) to ensure familiarity with the task. Participants were highly accurate during practice trials (mean accuracy: 91%), indicating comprehension of the task.

### Statistical analyses

For all statistical tests, alpha level of *p* < 0.05 was used to assess significance, tests were two-tailed, and we applied Bonferroni correction for multiple-comparisons where appropriate. All analyses were conducted in the R statistical programming environment^59^. Effect sizes were calculated using the *effectsize* package^60^. For each task (naturalistic/classic), we built a linear mixed-effects model to evaluate the predictivity of condition (low, medium, or high set size) on reaction time (RT) using the *lme4* package^61^. In each model, we included a fixed effect of condition. Additionally, we included a within-subject random effect of condition to account for individual variation in a) baseline reaction times (random intercepts) and b) individual efficiency (random slopes). Thus, we were able to separately estimate group-level and subject-level effects of the impact of condition on RT.

## Results

To investigate whether classic findings of visual search extend to naturalistic settings, we developed a novel paradigm in which participants searched for real-world objects inside of 360° real-world scenes. For each visual search task (naturalistic/classic), we evaluated the extent to which condition (low, medium, or high clutter/set size) predicts reaction times (RT) using a linear mixed-effects model. We hypothesized that greater set sizes would result in slower RTs in each task, and that individual estimates of this effect of set size on RT (search efficiency) would correlate across tasks (naturalistic / classic).

### Naturalistic visual search performance

**Figure 3:**
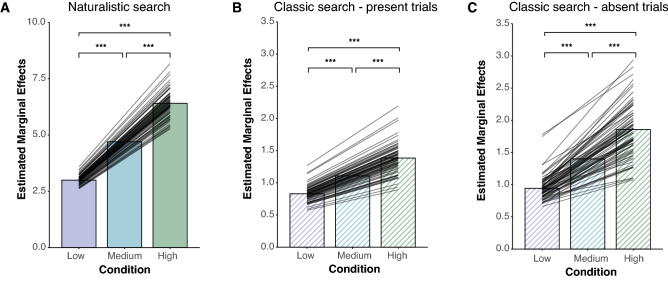
Experimental results. (**A**) Visual clutter modulates reaction times in real-world environments (*F*_(2,368.76)_ = 187.42, *p* < 0.001). Set size modulates reaction times on both (**B**) present trials (*F*_(2,116.08)_ = 463.04, *p* < 0.001) and (**C**) absent trials (*F*_(2,105.09)_ = 309.66, *p* < 0.001) in a classic visual search task. Lines indicate random slope fits for each participant. *p < 0.05, **p < 0.01, ***p < 0.001, n.s. *p* > 0.05.

We first examined the relationship between visual clutter levels and search performance inside immersive, real-world scenes. As predicted, we found that participants were faster and more accurate to locate the target in less-cluttered as compared with more-cluttered scenes. Combining data across our participants, we found a significant correlation between clutter-level and reaction times to correctly detect a target (*r*_*s*_ = 0.595, *p* < 0.001). This correlation was significant in all three sections of the environment: left, front, and right of the participant (left frame: *r*_*s*_ = 0.62, *p* < 0.001; front frame: *r*_*s*_ = 0.74, *p* < 0.001; right frame: *r*_*s*_ = 0.51, *p* = 0.032). Importantly, a one-way ANOVA on the fixed effect of clutter revealed a significant main effect on reaction times across participants (Fig. 3A; *F*_(2,368.76)_ = 187.42, *p* < 0.001, η_p_^2^ = 0.5). An additional one-way ANOVA demonstrated a main effect of condition on individual participant false alarm rate (*F*_(2,222)_ = 63.1, *p* < 0.001, η_p_^2^ = 0.36). Overall, these results suggest that visual clutter modulates visual search performance inside real-world scenes.

### Classic visual search performance

We next evaluated the relationship between set size and search performance in a classic visual search paradigm. For target present and target absent trials, we used separate linear mixed-effects models to evaluate the fixed effect of set size on RT while accounting for the random effect of subject. A one-way ANOVA conducted on the fixed effect of set size revealed a significant main effect of set size on RT across participants for both target present (Fig. 3B; *F*_(2,116.08)_ = 463.04, *p* < 0.001, η_p_^2^ = 0.89) and target absent trials (Fig. 3C; *F*_(2,105.09)_ = 309.66, *p* < 0.001, η_p_^2^ = 0.85). A separate one-way ANOVA demonstrated a main effect of set size on individual participant false alarm rates (*F*_(2,222)_ = 6.40, *p* = 0.002, η^2^ = 0.05). In sum, these results dovetail with previous findings of classic visual search paradigms that demonstrate the impact of set size on visual search performance^4^.

### Reliability of search efficiency

Before examining the relationship between performance on the two experimental paradigms, we established the reliability of search efficiency: the impact of set size on a participant’s RT. For each task, we split each participant’s RTs in half within each level of set size. We next fit a linear mixed effects model for each half-split of RT to estimate search efficiency, the random slope of condition for each participant. We calculate reliability (ρ*) as the Pearson’s correlation between search efficiency of one half and the other, corrected with the Spearman-Brown prediction formula to estimate the full-length task reliability. We find low reliability for naturalistic search efficiency (ρ* = 0.293) and high reliability for classic search efficiency on both target present (ρ* = 0.947) and target absent trials (ρ* = 0.947).

### Relating performance on naturalistic and classic visual search tasks

Having established the reliability of search efficiency within each task, we next investigated the relationship of search performance between the two tasks. For each task, we used a linear-mixed effects model to derive search efficiency: the random slope of condition fit to each participant’s RT. Importantly, we accounted for variability of RT within each task by modelling random intercepts for each participant.

**Figure 4:**
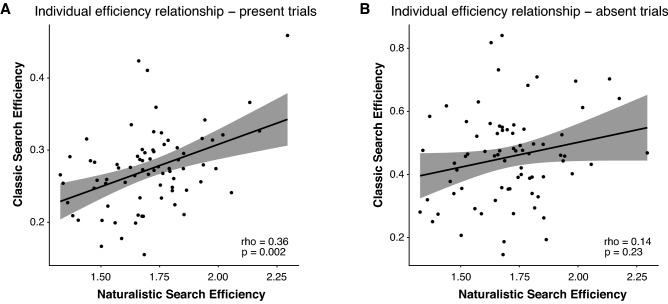
Relating individual performance across tasks. An individual’s efficiency in the classic visual search task relates to efficiency in the naturalistic visual search task for (**A**) target present trials (*r*_*s*_ = 0.36, *p* = 0.002) but not for (**B**) target absent trials (*r*_*s*_ = 0.14, *p* = 0.23).

We found a significant relationship between search efficiency on the naturalistic search task and on target present trials of the classic visual search task (Fig. 4A: *r*_*s*_ = 0.36, *p* = 0.002). However, the relationship between naturalistic and classic visual search was attenuated on target absent trials (Fig. 4B: *r*_*s*_ = 0.14, *p* = 0.23). We next compared individual efficiency in each quadrant (left, front, right) of the naturalistic visual search task with each trial type of the classic visual search task. Interestingly, efficiency in the front quadrant of the naturalistic visual search task was significantly related to efficiency on both target present and target absent trials (present: *r*_*s*_ = 0.27, *p* = 0.02; absent: *r*_*s*_ = 0.29, *p* = 0.012). While we also observed a significant relationship between efficiency in the right quadrant and target present trials (*r*_*s*_ = 0.28, *p* = 0.012), this relationship did not hold when considering target absent trials (*r*_*s*_ = 0.02, *p* = 0.85). Furthermore, we found no relationship between efficiency in the left quadrant and either classic search trial type (present: *r*_*s*_ = 0.15, *p* = 0.19; absent: *r*_*s*_ = 0.09, *p* = 0.46). Together, these results suggest that efficiency on a classic visual search task, indexed by a set size manipulation, predicts efficiency in naturalistic visual search, indexed by a clutter manipulation in complex, visual scenes.

## Discussion

We find that visual search in immersive, real-world environments bears remarkable similarities to classic search in two important senses. First, classic and naturalistic search performance are both limited by set size: just as classic search efficiency is limited by the number of distractors in the visual display, naturalistic search efficiency is limited by a real-world analogue of set size, visual clutter. Second, individual differences in search efficiency are related in both tasks: participants with steeper costs of set size in artificial arrays of letters were more severely impacted by visual clutter in real-world environments. Together, these findings suggest that classic search is an excellent model of search efficiency within real-world environments.

Relating individual performance between computer-based and naturalistic settings is central to identifying the cognitive factors and task strategies that facilitate visual search^3,62^. Differences in visual search performance have been demonstrated across development and healthy aging^63,64^, clinical diagnoses^65,66^, and expertise^67^. Further, the task of visual search is highly relevant to performance in various professional settings (e.g., radiology, airport security)^34–37,68^. For instance, previous research has shown a relationship between airport security officers’ search performance on a computer-based app and their detection of violations at an airport checkpoint, where people with faster and more accurate search within the app were better at detecting prohibited items at TSA checkpoints^40^. While studies within professional settings begin to establish connections between computer-based paradigms and naturalistic experience, both the examined populations (e.g., experts) and sampled contexts (e.g., TSA checkpoints) limit the generalizability of these results to diverse, real-world environments.

Advances in virtual reality (VR) technology present a promising avenue to investigate visual behavior within naturalistic stimuli and contexts while simultaneously maintaining experimental control^42–44^. First, VR enables researchers to exact similar rigor as in computer-based studies (e.g., trial length) without placing physical limitations (e.g., head-restraint) on the complex repertoire of participants’ naturalistic behavior. Second, researchers can leverage VR to efficiently investigate behavior across a wide range of diverse settings (e.g., beaches, parks, libraries), likely increasing the real-world generalizability of findings. Taken together, the use of VR empowers researchers to construct more representative models of naturalistic experience. Accordingly, an increasing number of studies employ VR headsets to investigate visual functions, providing essential connections between computer-based findings and naturalistic behavior. Yet, few studies have sought to relate models of visual functions, such as visual search, that are derived from behavior measured in traditional, computer-based paradigms to analogous behavior measured in real-world settings.

Recent studies investigating visual search using head-mounted displays highlight, in particular, that active behavior recruits memory to aid search performance in naturalistic settings. Active exploration of virtual environments prior to search has been shown to improve search performance by engaging spatial memory^46,69^, a benefit not seen for explicit memorization^70^. For example, one study demonstrates that spatial memory aids search by restricting attention to relevant areas of the scene^47^: when the location of a target object was changed from a learned location, participants continued to initially fixate on the learned location. Further, interaction with objects in virtual environments bolsters memory of target object locations: participants are faster to locate objects they arranged within a room compared with objects arranged by others^45^. By utilizing VR to investigate visual search, these studies reveal the contributions of action in and interaction with virtual environments on search performance. Our results extend prior research on active search by generalizing the well-known set size effect to a diverse set of real-world scenes, and by demonstrating a predictive relationship between an individual’s search efficiency in artificial and naturalistic contexts.

Certainly, our experimental paradigm has shortcomings. First, in contrast to many studies of visual search in which eye-tracking measures are employed, we were only able to use a combination of head-tracking data and keypress reaction times. This method is undoubtably noisier than measuring eye-tracking reaction times in each task. However, given the close coupling of head and eye movements^71^ and the presence of set size effects within both paradigms, we do not believe a different measurement would drastically alter our results. Second, while the classic search paradigm demonstrated high split-half reliability, the naturalistic search paradigm exhibited relatively low split-half reliability. Despite this low reliability, our results show a relationship of an individual’s search efficiency between the two visual search tasks. We hypothesize that, the magnitude of the task relationship would increase with more naturalistic search trials. Future studies are needed to test this hypothesis, as well as to understand behavioral changes across a continuum of stimulus naturalism moving from well-controlled psychophysical displays to naturalistic settings.

In sum, we find that set size analogously limits visual search performance in both classic, computer-based visual search and immersive, real-world scenes. Further, individual search efficiency on a classic search task predicts search efficiency in a naturalistic search task. These findings suggest that individual search performance is limited by common properties in artificial and naturalistic contexts and have important implications for relating models of vision to real-world behavior.

## Supplementary Information

Supplementary Information.

## Data Availability

Requests for materials should be directed to T.L.B.

## References

[1] Treisman AM, Gelade G (1980). A feature-integration theory of attention. Cognit. Psychol..

[2] Wolfe JM, Võ ML-H, Evans KK, Greene MR (2011). Visual search in scenes involves selective and nonselective pathways. Trends Cogn. Sci..

[3] Wolfe JM (2020). Visual search: How do we find what we are looking for?. Annu. Rev. Vis. Sci..

[4] Wolfe JM, Horowitz TS (2017). Five factors that guide attention in visual search. Nat. Hum. Behav..

[5] Wolfe JM (1994). Guided search 2.0 a revised model of visual search. Psychon. Bull. Rev..

[6] Geisler WS, Cormack LK (2011). Models of Overt Attention.

[7] Eckstein MP (2011). Visual search: A retrospective. J. Vis..

[8] Lindsay GW (2020). Attention in psychology, neuroscience, and machine learning. Front. Comput. Neurosci..

[9] Peelen MV, Kastner S (2014). Attention in the real world: Toward understanding its neural basis. Trends Cogn. Sci..

[10] Anderson BA (2016). Social reward shapes attentional biases. Cogn. Neurosci..

[11] Maunsell JHR (2004). Neuronal representations of cognitive state: Reward or attention?. Trends Cogn. Sci..

[12] Rust NC, Cohen MR (2022). Priority coding in the visual system. Nat. Rev. Neurosci..

[13] Henderson J (2003). Human gaze control during real-world scene perception. Trends Cogn. Sci..

[14] Tatler BW, Hayhoe MM, Land MF, Ballard DH (2011). Eye guidance in natural vision: Reinterpreting salience. J. Vis..

[15] Hayhoe, M. M. Vision and Action. 27 (2017). 10.1146/annurev-vision-102016-061437 28715958

[16] Biederman I, Glass AL, Stacy EW (1973). Searching for objects in real-world scenes. J. Exp. Psychol..

[17] Potter MC (1975). Meaning in visual search. Science.

[18] Võ ML-H, Wolfe JM (2012). When does repeated search in scenes involve memory? Looking at versus looking for objects in scenes. J. Exp. Psychol. Hum. Percept. Perform..

[19] Võ ML-H, Wolfe JM (2013). The interplay of episodic and semantic memory in guiding repeated search in scenes. Cognition.

[20] Castelhano MS, Heaven C (2010). The relative contribution of scene context and target features to visual search in scenes. Atten. Percept. Psychophys..

[21] Castelhano MS, Henderson JM (2007). Initial scene representations facilitate eye movement guidance in visual search. J. Exp. Psychol. Hum. Percept. Perform..

[22] Draschkow D, Kallmayer M, Nobre AC (2021). When natural behavior engages working memory. Curr. Biol..

[23] Draschkow D, Nobre AC, van Ede F (2022). Multiple spatial frames for immersive working memory. Nat. Hum. Behav..

[24] Felsen G, Dan Y (2005). A natural approach to studying vision. Nat. Neurosci..

[25] Leopold DA, Park SH (2020). Studying the visual brain in its natural rhythm. Neuroimage.

[26] Wolfe JM (2016). Rethinking the basic-applied dichotomy. Cogn. Res. Princ. Implic..

[27] Brunyé TT, Drew T, Weaver DL, Elmore JG (2019). A review of eye tracking for understanding and improving diagnostic interpretation. Cogn. Res. Princ. Implic..

[28] Wolfe JM (2016). Use-inspired basic research in medical image perception. Cogn. Res. Princ. Implic..

[29] Clark K, Cain MS, Adamo SH, Mitroff SR, Dodd MD, Flowers JH (2012). Overcoming hurdles in translating visual search research between the lab and the field. The Influence of Attention, Learning, and Motivation on Visual Search.

[30] Blacker KJ, Peltier C, McKinley RA, Biggs AT (2020). What versus how in visual search: Effects of object recognition training, strategy training, and non-invasive brain stimulation on satellite image search. J. Cogn. Enhanc..

[31] See JE, Drury CG, Speed A, Williams A, Khalandi N (2017). The role of visual inspection in the 21st century. Proc. Hum. Factors Ergon. Soc. Annu. Meet..

[32] Drew T, Williams LH, Aldred B, Heilbrun ME, Minoshima S (2018). Quantifying the costs of interruption during diagnostic radiology interpretation using mobile eye-tracking glasses. J. Med. Imaging.

[33] van der Gijp A (2017). How visual search relates to visual diagnostic performance: A narrative systematic review of eye-tracking research in radiology. Adv. Health Sci. Educ..

[34] Adamo SH, Ericson JM, Nah JC, Brem R, Mitroff SR (2018). Mammography to tomosynthesis: examining the differences between two-dimensional and segmented-three-dimensional visual search. Cogn. Res. Princ. Implic..

[35] Biggs AT, Cain MS, Clark K, Darling EF, Mitroff SR (2013). Assessing visual search performance differences between Transportation Security Administration Officers and nonprofessional visual searchers. Vis. Cogn..

[36] Mendes M, Schwaninger A, Michel S (2013). Can laptops be left inside passenger bags if motion imaging is used in X-ray security screening?. Front. Hum. Neurosci..

[37] Clancy Dollinger SM (1994). Individual differences in visual search performance among medical technologists. Personal. Individ. Differ..

[38] Evans KK, Georgian-Smith D, Tambouret R, Birdwell RL, Wolfe JM (2013). The gist of the abnormal: Above-chance medical decision making in the blink of an eye. Psychon. Bull. Rev..

[39] Williams LH, Drew T (2019). What do we know about volumetric medical image interpretation?: A review of the basic science and medical image perception literatures. Cogn. Res. Princ. Implic..

[40] Mitroff SR, Ericson JM, Sharpe B (2018). Predicting airport screening officers’ visual search competency with a rapid assessment. Hum. Fact..

[41] Haskins AJ, Mentch J, Botch TL, Robertson CE (2020). Active vision in immersive, 360° real-world environments. Sci. Rep..

[42] Doucet G, Gulli RA, Martinez-Trujillo JC (2016). Cross-species 3D virtual reality toolbox for visual and cognitive experiments. J. Neurosci. Methods.

[43] Scarfe P, Glennerster A (2015). Using high-fidelity virtual reality to study perception in freely moving observers. J. Vis..

[44] Draschkow D (2022). Remote virtual reality as a tool for increasing external validity. Nat. Rev. Psychol..

[45] Draschkow D, Võ ML-H (2017). Scene grammar shapes the way we interact with objects, strengthens memories, and speeds search. Sci. Rep..

[46] Beitner J, Helbing J, Draschkow D, Võ ML-H (2021). Get your guidance going: Investigating the activation of spatial priors for efficient search in virtual reality. Brain Sci..

[47] Li C-L, Aivar MP, Tong MH, Hayhoe MM (2018). Memory shapes visual search strategies in large-scale environments. Sci. Rep..

[48] Marek N, Pollmann S (2020). Contextual-cueing beyond the initial field of view—A virtual reality experiment. Brain Sci..

[49] Neider MB, Zelinsky GJ (2008). Exploring set size effects in scenes: Identifying the objects of search. Vis. Cogn..

[50] Palmer J (1994). Set-size effects in visual search: The effect of attention is independent of the stimulus for simple tasks. Vis. Res..

[51] Henderson JM, Chanceaux M, Smith TJ (2009). The influence of clutter on real-world scene search: Evidence from search efficiency and eye movements. J. Vis..

[52] Wolfe JM, Alvarez GA, Rosenholtz R, Kuzmova YI, Sherman AM (2011). Visual search for arbitrary objects in real scenes. Atten. Percept. Psychophys..

[53] Bar M (2004). Visual objects in context. Nat. Rev. Neurosci..

[54] Võ ML-H, Boettcher SE, Draschkow D (2019). Reading scenes: How scene grammar guides attention and aids perception in real-world environments. Curr. Opin. Psychol..

[55] Rosenholtz R, Li Y, Nakano L (2007). Measuring visual clutter. J. Vis..

[56] Kravitz DJ, Peng CS, Baker CI (2011). Real-world scene representations in high-level visual cortex: It’s the spaces more than the places. J. Neurosci..

[57] Lee, J. H., Han, M.-K., Ko, D. W. & Suh, I. H. From Big to Small: Multi-Scale Local Planar Guidance for Monocular Depth Estimation. *ArXiv190710326 Cs* (2020).

[58] Yu C-P, Samaras D, Zelinsky GJ (2014). Modeling visual clutter perception using proto-object segmentation. J. Vis..

[59] R Core Team. R: A language and environment for statistical computing (2013).

[60] Ben-Shachar M, Lüdecke D, Makowski D (2020). Effectsize: Estimation of effect size indices and standardized parameters. J. Open Source Softw..

[61] Bates, D., Mächler, M., Bolker, B. & Walker, S. Fitting linear mixed-effects models using lme4. *J. Stat. Softw.* **67** (2015).

[62] Biggs AT, Kramer MR, Mitroff SR (2018). Using cognitive psychology research to inform professional visual search operations. J. Appl. Res. Mem. Cogn..

[63] Hommel B, Li KZH, Li S-C (2004). Visual search across the life span. Dev. Psychol..

[64] Woods AJ (2013). The development of organized visual search. Acta Psychol. (Amst.).

[65] Plaisted K, O’Riordan M, Baron-Cohen S (1998). Enhanced visual search for a conjunctive target in autism: A research note. J. Child Psychol. Psychiatry.

[66] O’Riordan MA, Plaisted KC, Driver J, Baron-Cohen S (2001). Superior visual search in autism. J. Exp. Psychol. Hum. Percept. Perform..

[67] Abernethy B, Russell DG (1987). The relationship between expertise and visual search strategy in a racquet sport. Hum. Mov. Sci..

[68] Lanagan-Leitzel LK, Skow E, Moore CM (2015). Great expectations: Perceptual challenges of visual surveillance in lifeguarding: Visual surveillance in lifeguarding. Appl. Cogn. Psychol..

[69] Li C-L, Aivar MP, Kit DM, Tong MH, Hayhoe MM (2016). Memory and visual search in naturalistic 2D and 3D environments. J. Vis..

[70] Helbing J, Draschkow D, Võ ML-H (2020). Search superiority: Goal-directed attentional allocation creates more reliable incidental identity and location memory than explicit encoding in naturalistic virtual environments. Cognition.

[71] Freedman EG (2008). Coordination of the eyes and head during visual orienting. Exp. Brain Res..

