# Supplementary material for: Active visual search in naturalistic environments reflects individual differences in classic visual search performance
Source: Sci Rep. 2023 Jan 12;13:631. doi: 10.1038/s41598-023-27896-7 (PMC9837148; doi:10.1038/s41598-023-27896-7)
Supplement: Supplementary file 1 — Supplementary Information. [file 41598_2023_27896_MOESM1_ESM.docx]

**Active visual search in naturalistic environments reflects individual differences in classic visual search performance**

Thomas L. Botch^1*^, Brenda D. Garcia^1^, Yeo Bi Choi^1^, Nicholas Feffer^2,3^, Caroline E. Robertson^1^

^1^ *Department of Psychological and Brain Sciences, Dartmouth College, Hanover, NH 03755, USA*

^2^ *Department of Computer Science, Dartmouth College, Hanover, NH 03755, USA*

^3^ *Department of Computer Science, Stanford University, Stanford, CA 94305, USA*

* Corresponding author: *Thomas L. Botch, thomas.l.botch@dartmouth.edu*

**Supplemental Information**

**Supplemental Video S1** [**https://youtu.be/C3puo0vIqbo**](https://youtu.be/C3puo0vIqbo)**.** Example naturalistic visual search trial. Participants were required to fixate at world center for 3s before being presented with a conjunctive word description (e.g., the yellow car) of the target object. Subsequently, participants actively searched for the described object inside an immersive photosphere.
